# Elucidating cryptic dynamics of *Theileria* communities in African buffalo using a high‐throughput sequencing informatics approach

**DOI:** 10.1002/ece3.5758

**Published:** 2019-12-20

**Authors:** Caroline K. Glidden, Anson V. Koehler, Ross S. Hall, Muhammad A. Saeed, Mauricio Coppo, Brianna R. Beechler, Bryan Charleston, Robin B. Gasser, Anna E. Jolles, Abdul Jabbar

**Affiliations:** ^1^ Department of Integrative Biology Oregon State University Corvallis OR USA; ^2^ Department of Veterinary Biosciences Melbourne Veterinary School University of Melbourne Werribee Vic. Australia; ^3^ Carlson College of Veterinary Medicine Oregon State University Corvallis OR USA; ^4^ The Pirbright Institute Woking UK

**Keywords:** disease ecology, high‐throughput amplicon sequencing, next‐generation sequencing, parasite community ecology

## Abstract

1. Increasing access to next‐generation sequencing (NGS) technologies is revolutionizing the life sciences. In disease ecology, NGS‐based methods have the potential to provide higher‐resolution data on communities of parasites found in individual hosts as well as host populations.
2. Here, we demonstrate how a novel analytical method, utilizing high‐throughput sequencing of PCR amplicons, can be used to explore variation in blood‐borne parasite (*Theileria*—Apicomplexa: Piroplasmida) communities of African buffalo at higher resolutions than has been obtained with conventional molecular tools.
3. Results reveal temporal patterns of synchronized and opposite fluctuations of prevalence and relative abundance of *Theileria* spp. within the host population, suggesting heterogeneous transmission across taxa. Furthermore, we show that the community composition of *Theileria* spp. and their subtypes varies considerably between buffalo, with differences in composition reflected in mean and variance of overall parasitemia, thereby showing potential to elucidate previously unexplained contrasts in infection outcomes for host individuals.
4. Importantly, our methods are generalizable as they can be utilized to describe blood‐borne parasite communities in any host species. Furthermore, our methodological framework can be adapted to any parasite system given the appropriate genetic marker.
5. The findings of this study demonstrate how a novel NGS‐based analytical approach can provide fine‐scale, quantitative data, unlocking opportunities for discovery in disease ecology.

## Graphical abstract

We used high‐throughput amplicon sequencing to uncover cryptic variation in blood‐borne parasite communities of African buffalo. Our methods are generalizable and will unlock opportunity for discovery in disease ecology.

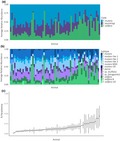

## 1 INTRODUCTION

The increasing availability of NGS data is revolutionizing many aspects of the life sciences—from novel insights into microbial ecology and microbiomes (Costello et al., 2009) to innovative monitoring tools for biodiversity (Smith, Thomas, Levi, Wang, & Wilmers, 2018; Yoccoz, 2012). In disease ecology, NGS‐based analytical methods have the potential to provide higher‐resolution data on communities of parasites found in individual hosts and host populations than data obtained using conventional diagnostic approaches (highlighted in Zylberberg, 2019).

Until now, disease ecologists have used standard medical and/or veterinary diagnostic approaches to detect parasites, including microscopy (Hernandez‐Lara, Gonzalez‐Garcia, & Santiago‐Alarcon, 2017; Jolles, Ezenwa, Etienne, Turner, & Olff, 2008), antibody‐based techniques (Beechler et al., 2015; Gorsich et al., 2018), and conventional polymerase chain reaction (PCR) (Telfer et al., 2010). These approaches often suffer, to varying degrees, from three limitations that are relevant for investigating parasites in ecological systems: (a) a lack of breadth, (b) a lack of depth, and/or (c) lack of precision. (a) With tools that require specific binding agents for each taxon of interest (e.g., PCR‐ and antibody‐based detection), researchers typically only detect the specific parasites or parasite groups that the diagnostic assay is designed for. In particular, in the context of nonmodel host organisms and emerging infectious diseases, researchers may not detect parasites that are key drivers of community dynamics and/ or novel parasites. Nonspecific methods can ensure broad detection of etiological agents (Glidden et al., 2018); however, (b) identifying important within‐host interactions (between parasite and host immune response and/or other parasites) may necessitate identifying infectious agents at low taxonomic levels. For example, infections by *Plasmodium*, the etiological agent of malaria in humans, often consist of multiple genotypes (Arnot, 1998; Smith, Felger, Tanner, & Beck, 1999). *Plasmodium* genotypes can respond differentially to treatments, with particular genotypes resistant to antimalarial medication (Huijben, Sim, Nelson, & Read, 2011). In the absence of treatment, drug‐resistant strains are suppressed by their nonresistant counterparts; however, treatment results in a “competitive release” of drug‐resistant genotypes (Huijben et al., 2011), which could result in higher prevalence and abundance of drug‐resistant parasites within a host population. (c) Finally, when analyzing the impact of parasite interactions on parasite transmission and host health, presence/absence data (as opposed to abundance or relative abundance) may mask intricate community interactions (Budischack et al., 2018; Lello, Boag, Fenton, Stevenson, & Hudson, 2004). Consequently, conventional diagnostic approaches often fail to capture variation in parasite community structure that is relevant to understanding parasite transmission dynamics and differential infection outcomes for the host.

Promisingly, novel molecular techniques are increasing disease ecologists' capacity to evaluate the structure and dynamics of parasite communities across a range of taxonomic scales. NGS of amplicons (NGSA, hereafter) can magnify the information obtained in one assay (Ogorzaly et al., 2015), as this approach targets one region of DNA and provides millions of sequences with low error rates (Glenn, 2011). Primers designed to target DNA can be conserved across high taxonomic levels, while encompassing enough nucleotide variation to distinguish among species or genotypes, enabling simultaneous detection of a multitude of taxa (Lindahl et al., 2013), and an estimation of the relative abundance of each taxon within a sample (Nelson, Morrison, Benjamino, Grim, & Graf, 2014). NGSA rose to popularity through microbiome research, which uses NGSA to target a short segment of the 16S rRNA gene to describe highly diverse microbial communities (Costello et al., 2009). Recently, NGSA has been used to identify diversity in micro‐ and macroparasite communities in the rufous mouse lemur (*Microcebus rufus*) (Aivelo & Norberg, 2018), *Trypanosoma* communities in the koala (*Phascolarctos cinereus*) (Barbosa et al., 2017), and *Eimeria* communities in the brush‐tailed rock‐wallaby (*Petrogale penicillata*) (Vermeulen, Lott, Eldridge, & Power, 2016). Along with the latest developments in NGSA technologies, new bioinformatic tools (such as SeekDeep; Hathaway, Parobek, Juliano, & Bailey, 2017) have enabled the detection of variation down to a single nucleotide level in Illumina MiSeq data, thereby allowing for the distinction between or among haplotypes/subtypes (e.g., *Plasmodium* spp.; Boyce et al., 2018; Hathaway et al., 2017; Zhong et al., 2018).

**Figure 1:**
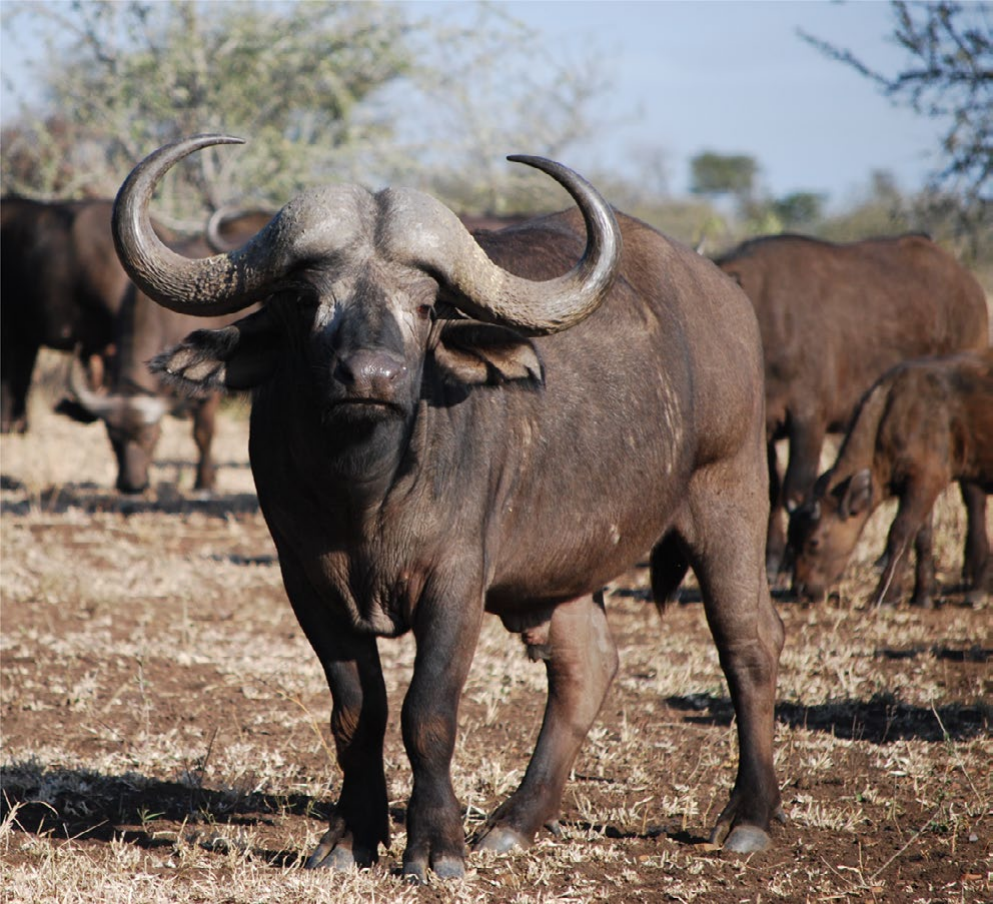
African buffalo in Kruger National Park, South Africa. Photograph taken by Robert Spaan

Here, we used NGSA and SeekDeep to obtain qualitative and quantitative sequence data for piroplasm communities, at the species clade and subtype levels, in a herd of African buffalo (Figure 1) caught every 2–3 months for 2 years. Piroplasms are intracellular protists, including the genera *Theileria* and *Babesia*, which infect the red and/or white blood cells of a range of host species (Abdela & Tilahun, 2016; Homer, Aguilar‐Delfin, Telford, Krause, & Persing, 2000; Tarav et al., 2017; Yabsley & Shock, 2013). Piroplasms are particularly important parasite species within eastern and southern African ecosystems, as infections can cause substantial mortality in wildlife of conservation concern (Nijhof et al., 2005), as well as mortality and decreased productivity in economically significant livestock (Schoeman, 2009). Although rarely infected with *Babesia* spp. (Henrichs et al., 2016; Mans, Pienaar, Ratabane, Pule, & Latif, 2016), African buffalo have been reported to be simultaneously infected with multiple species of *Theileria*, encompassing a multitude of subtypes (Mans, Pienaar, & Latif, 2015). Infection with multiple *Theileria* spp., as opposed to single‐species infections, results in dramatically different pathological disorders in cattle (Woolhouse et al., 2015), indicating that parasite interactions can adversely impact host health.

Disentangling the complex African buffalo—*Theileria* system poses two major challenges that previous studies using conventional approaches were unable to overcome: First, *Theileria* is taxonomically complex and classical taxonomists have difficulty distinguishing between species and haplotypes, requiring genetically distinct, yet closely related organisms, to remain distinguished as “subtypes” (reviewed in Mans et al., 2015). Importantly, subtypes are restricted by host specificity and geographic range, indicating important biological differences (Chaisi, Collins, Potgieter, & Oosthuizen, 2013; Mans, Pienaar, & Latif, 2011; Mans et al., 2016; Pienaar, Potgieter, Latif, Thekisoe, & Mans, 2011). Attempting to differentiate between subtypes using PCR‐ and antibody‐based approaches has been riddled with issues of crossreactivity (Mans et al., 2015). Second, *Theileria* spp. are too common in African buffalo for presence/absence data to be useful in understanding disease dynamics (i.e., animals are almost always infected with all species; Henrichs et al., 2016). Thus, uncovering *Theileria* community dynamics necessitates quantitative data at fine‐scale taxonomic resolution, making the African buffalo—*Theileria* system an ideal case study for describing the power of NGSA techniques in disease ecology.

We demonstrate how combining NGSA and novel bioinformatic tools enables a sound estimation of parasite transmission and persistence dynamics by describing population prevalence (i.e., the number of hosts infected) and population frequency (the relative abundance of each taxon in the host system) of each taxon. We then demonstrate how our methods can be used to evaluate the effect of community dynamics on infection outcome by assessing variation in *Theileria* communities among hosts and showing that variation in community structure relates to parasitemia—a proxy for the magnitude of the effect of parasites on host health (e.g., Asghar, Hasselquist, & Bensch, 2011; Sol, Jovani, & Torres, 2003; Stjernman, Raberg, & Nilsson, 2008). Overall, we highlight how novel molecular and bioinformatic techniques can provide the breadth, depth, and precision of data needed to understand parasite community dynamics within host populations and in individual hosts.

## 2 METHODS

### 2.1 Study site

African buffalo included in this study were located in a 900‐ha enclosure within the Kruger National Park (KNP) a 19,000‐km^2^ preserve, located in northeastern South Africa (S 24 23′ 52″, E 31 46′ 40″). The enclosure is entirely within KNP and has numerous other wild animals typical of the ecosystem (e.g., giraffe, zebra, warthogs, small mammals, and small predators). However, the enclosure excludes megaherbivores (rhino, hippo, elephant) and large predators (lion, leopard). Study animals graze and breed naturally and find water in seasonal pans and man‐made (permanent) water troughs. In extreme dry seasons, supplemental grass and alfalfa hay is supplied.

### 2.2 Sample collection and DNA extraction

A herd of 41–54 individually marked buffalo, of varying sex and age, was maintained throughout this study. Natural births and deaths occurred, leading to a total of 66 individuals sampled for this study and 443 samples. Buffalo were captured every two to three months from February 2014 to October 2015, totaling nine sampling time points. Animals were included in the study if they were captured at least two times. Animal capture and sedation protocols have previously been described by Glidden et al. (2018). During each capture, 2 ml of whole blood was collected via jugular venipuncture directly into EDTA‐coated vacutainers and stored on ice during transport. One milliliter of whole blood was pipetted into sterile microcentrifuge tubes and stored at −80°C until used for DNA extractions while the rest of blood was immediately used to measure red blood cell counts using an automated hematology analyzer (Vet ABC, Scil Animal Care Company).

DNA was extracted from 200 µl of EDTA blood using DNeasy Blood and Tissue Kit (Qiagen) following the manufacturer's protocol. DNA extractions were shipped to the University of Melbourne, Australia, and stored at −20°C until further testing.

### 2.3 Next‐generation sequencing of PCR amplicons

#### 2.3.1 Library preparation and Illumina MiSeq

The V4 hypervariable fragment (~500 bp) of the 18S rRNA gene of *Theileria* was targeted for the NGSA. Briefly, PCR amplicons were generated using the RLBF (5′–GAG GTA GTG ACA AGA AAT AAC AAT–A3′) and RLBR (5′–TCT TCG ATC CCC TAA CTT TC–3′) primers (Gubbels et al., 1999) using the AmpliTaq Gold 360 mastermix (Life Technologies) in a thermal cycler (Veriti‐384™; Applied Biosystem). The first PCR was run for the initial denaturation for 2 min at 94°C followed by 30 cycles of 30 s at 94°C, 30 s at 57°C, and 1 min at 72°C and a final extension of 8 min at 72°C. PCR amplicons were purified using magnetic beads and visualized on 2% E‐Gel Agarose Gel stained with SYBR Safe DNA Gel Stain (Thermo Fisher). The second PCR was performed to index the amplicons using the TaKaRa Taq DNA Polymerase (Clontech), and it was run for 2 min at 94°C, 15 cycles of 30 s at 94°C, 30 s at 57°C, 1 min at 72°C, and a final extension of 1 min at 72°C. The PCR products were then purified using magnetic beads, quantified by fluorometry (QuantiFlour® dsDNA System), and normalized. The equimolar pool of amplicons was cleaned again using magnetic beads to concentrate the pool and then measured using an Agilent High‐Sensitivity D1000 Tape System (Agilent Technologies). The pool was diluted to 5 nM, and the molarity was confirmed again using the Tape System and sequenced on an Illumina MiSeq Reagent Kit v3 (600 cycle) using 2 × 300 base pairs paired‐end reads. Positive (*Theileria orientalis*) and negative (no DNA template) controls were also included during each step of the experiment.

#### 2.3.2 Bioinformatic analyses

As this study aimed to describe community dynamics of closely related taxa, the objective of the bioinformatic analysis was to filter and cluster sequences with single base‐pair resolution and calculate relative abundance of each unique sequence within a sample.

DADA2 (run in Qiime2 V. 2016.6.0 using the DADA2 plugin: V. 2018.6.0; Callahan et al., 2016) and SeekDeep (V 2.5.1; Hathaway et al., 2017) are two filtering and clustering softwares reported to obtain single base‐pair resolution. To decide on the best pipeline to use for the analysis, an in silico mock *Theileria* community analysis was conducted to test reproducibility of each software (Appendix S1). After our mock community analysis, we decided to use SeekDeep for all analyses. Furthermore, 10% of our samples were run in duplicate. We confirmed repeatability up to 1% relative abundance and use this cutoff throughout the rest of our analyses (Appendix S2).

Subsequently, FASTQ files from all samples were processed using a within‐sample relative abundance cutoff of 1% and the Illumina MiSeq tag, allowing no mismatches. Within the SeekDeep pipeline, sequences that were marked as likely chimeric were removed. Additionally, we removed any sequences that occurred once within the study as this would imply a unique sequence that occurred in one animal at one time point. Phred quality score of each consensus sequence was assessed in FastQC (V. 0.11.7). As the final PCR amplicon is ~460 bp, sequences were retained in the analysis if bases had an average Phred quality score >30 (1 error per 1,000 bases).

#### 2.3.3 Phylogenetic analyses

Bayesian inference (BI) and neighbor joining (NJ) analyses were conducted to identify sequences. First, a nonredundant database of all *Theileria* and *Babesia* subtypes known to infect African buffalo, as well as closely related species, was curated using the existing literature (Mans et al., 2015) and the NCBI database (GenBank). SeekDeep sequences and reference sequences were imported into Mesquite (V 3.51; Maddison & Maddison, 2018) and aligned using MUSCLE (Edgar, 2004). For the BI analysis, the likelihood parameters were based on the Akaike Information Criterion (AIC) test in jModeltest V.2.1.10 (Darriba, Taboada, Doallo, & Posada, 2012; Guindon & Gascuel, 2003). The likelihood parameters used were TrN + I + G (Nst = 6; rates = invariable + gamma). A Bayesian tree was constructed using the Monte Carlo Markov Chain analysis in MrBayes (V.3.1.2). Four simultaneous tree‐building chains were used to calculate posterior probabilities for 2,000,000 generations, saving every 100th tree. A consensus tree was constructed based upon the final 75% of trees produced (burnin = 0.25%).

The NJ analyses were conducted in MEGA 7.0 (Kumar, Stecher, & Tamura, 2016), and the nodes were tested for robustness with 10,000 bootstrap replicates. The data format was set to DNA, and gaps were treated as missing data. For the substitution model, substitution type was nucleotide, the method used was the number of differences, substitutions included were transitions and transversions, and rates among sites were uniform.

The tree topology was checked for concordance. *Theileria* spp. clades were considered supported if NJ bootstrapping values were >75% and Bayesian posterior probability values were >0.95. Subtype clades were considered supported if NJ bootstrapping values were >75%.

### 2.4 Calculation of parasitemia

Quantitative methodology used to calculate parasitemia of the collective *Theileria* genus (i.e., community abundance), including development of a quantitative PCR, is outlined in Appendix S3.

### 2.5 Community composition

*R* software (version 3.4.3) was used for all *Theileria* community analyses.

#### 2.5.1 Population patterns

To evaluate patterns of *Theileria* communities across the host population and generate hypotheses regarding differences in taxon transmission, we calculated prevalence of each taxon over the study period (*Theileria* spp.‐positive samples/total number of samples), prevalence of each taxon at each sampling time point (*Theileria* spp. positive samples/total number of samples per sample collecting point), frequency of each taxon over the entire study (number of individual *Theileria* spp. sequences/total number of *Theileria* sequences), and frequency of each taxon at each sampling time point (number of sequences per *Theileria* spp./total number of *Theileria* sequences per time point).

#### 2.5.2 Community analyses: Individual patterns

To evaluate individual patterns of *Theileria* communities, we used PERMANOVA to assess whether communities were significantly different between individual animals, with communities characterized by relative abundance of taxa. PERMANOVA was run for clade and subtype communities. We ran PERMONVA using the adonis function in “vegan” (Oksanen et al., 2007), including individual ID and sampling time point as fixed effects. The community dissimilarity matrices were calculated using Bray–Curtis distance measures.

To explore the relationship between *Theileria* communities and infection outcome, we visualized variation in community composition (i.e., the presence and relative abundance of each taxon within a sample) in relation to mean (±standard error of mean) parasitemia for each animal. First, we calculated average relative abundance of each taxon, at the clade and subtype level per animal followed by mean (±*SE*) parasitemia per animal. Subsequently, average (±*SE*) parasitemia per animal was then plotted from highest to lowest. Stacked bar plots for average communities for each animal were plotted using “phyloseq” (McMurdie & Holmes, 2013) and “ggplot2” (Wickham, 2016).

## 3 RESULTS

### 3.1 NGS of PCR amplicons reveals diverse parasite communities

#### 3.1.1 Bioinformatic analyses

A total of 440 (of 443) DNA samples were amplified and sequenced. A total of 32,727,499 reads passed quality trimming, with an average of 69,407 ± 1,929 reads per sample. The median number of reads per sample was 60,285 (interquartile 1:48,127; interquartile 3:78,472). Three samples had less than 1,000 reads and were removed from further analyses while 17 unique sequences appeared in only one sample each and were removed from analyses. A total of 29 unique sequences were identified. Our negative control only very weakly amplified (read count = 77), and we did not find any sequences due to contamination.

On average, sequences were 455 (*SE* ± 0.59) nucleotides in length, ranging from 460 to 451 nucleotides.

#### 3.1.2 Phylogenetic analyses

**Figure 2:**
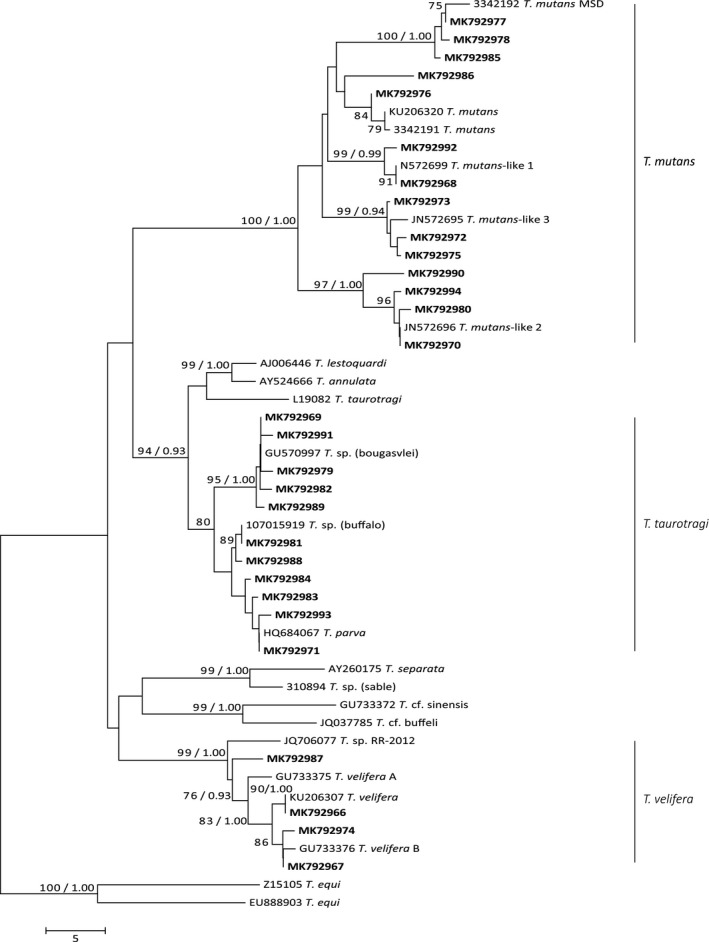
Phylogenetic relationship among consensus sequences of *Theileria* spp. determined in this study (bold) and the reference sequences for all *Theileria* spp. that infect African buffalo as well as closely related species (regular font, sequences with subtype names). Relationships were inferred from phylogenetic analysis of sequence data for a ~460‐bp region of the 18S V4 rRNA gene by neighbor joining and Bayesian inference. Neighbor joining bootstrap values >75% and Bayesian inference posterior probabilities >0.90 are included on tree branches

Bayesian inference and NJ phylogenetic methods produced trees with similar topologies; hence, only a representative NJ tree is presented here (Figure 2).

Analyses of 29 unique sequences revealed three main clades (Figure 2).

The first clade contained four sequences which grouped with previously published sequences of the *T. velifera* clade, with strong statistical support (bootstrap value of NJ = 99%; posterior probability value for BI = 1.0). One of these sequences (MK792966) grouped with *T. velifera* (KU206307), two (MK792967 and MK792974) with *T. velifera* B (GU33376), and one (MK792987) in between *T. velifera* A (GU733375) and *T. velifera*‐like sequence (JQ706077) (Figure 2).

The second clade contained 11 sequences and grouped within the *T. taurotagi* clade (nodal support NJ = 95%; BI = 0.93). Five sequences (MK792969, MK792979, MK792982, MK792989, and MK792991) grouped with *T*. sp. (*bougasvlei*) (nodal support NJ = 95%; BI = 1.0), whereas the remaining four (MK792971, MK792983, MK792984, and MK792993) and two (MK792981 and MK792988) grouped with *T. parva* and *T*. sp. (buffalo), respectively (Figure 2). The past literature has obtained similar bootstrap support for *T. parva* and *T*. sp. (buffalo) (Mans et al., 2015: nodal support NJ = 65%; Mans et al., 2011: NJ bootstrap value of 64); however, analysis using alternative markers has differentiated these as unique taxa (Bishop et al., 2015).

The third clade contained 14 sequences that grouped within the *T. mutans* clade (nodal support NJ = 100%; BI = 1.0). The *T. mutans* clade included six subtypes: *T. mutans‐*like 1 (MK792968, MK792992); *T. mutans*‐like 2 (MK792970, MK792980, MK792994, MK792990); *T. mutans‐*like 3 (MK792972, MK792973, MK792975); *T. mutans* MSD (MK792977, MK792978, MK792985); *T. mutans* (MK792976); and one in between *T. mutans* and *T. mutans* MSD (MK792986) (Figure 2). Pairwise differences (%), and prevalence and frequencies of 29 sequences are provided in Tables S1 and S2.

### 3.2 *Theileria* communities vary in time and across individuals

#### 3.2.1 Population patterns

We evaluated patterns of *Theileria* spp. infection in our study population averaged over the entire study period and change in infection patterns over time, by assessing the prevalence (number of samples the taxon appeared in/number of samples in study or at time step) and frequency (number of sequences per taxon/number of sequences in study or at time step) of *Theileria* clades and subtypes.

**Figure 3:**
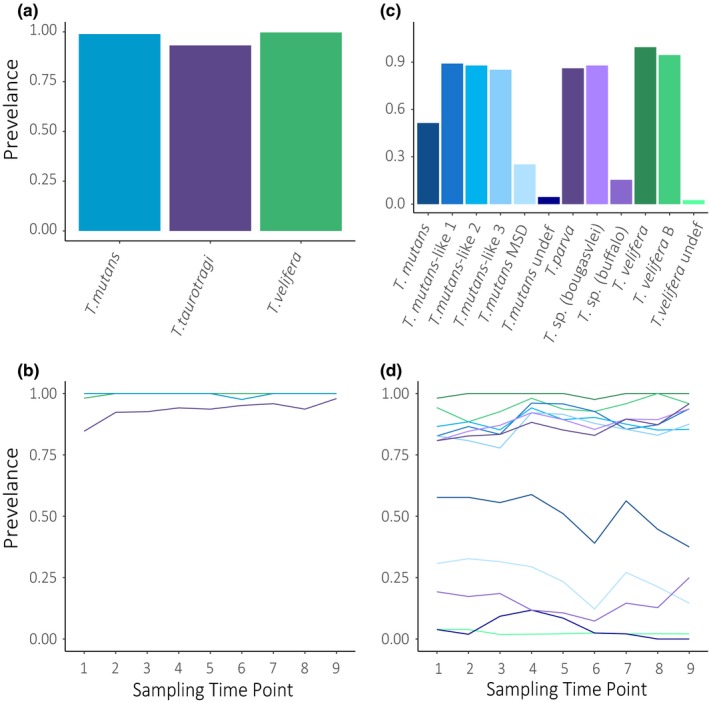
Prevalence of *Theileria* communities. The overall prevalence of *Theileria* spp. over the entire study and at each sampling time point for each clade (a, b) and subtype (c, d). Note: Colors for each taxon are identical in bar plots and line graphs

Clade‐level analysis of *Theileria* spp. prevalence suggested a uniform and time‐invariable high prevalence of all three clades (Figure 3a,b; Table S2). However, higher taxonomic resolution revealed variation in overall prevalence (Figure 3c; Table S2) as well as temporal variation in subtypes in the population (Figure 3d). For example, each *Theileria* clade contained 2–3 common subtypes (overall prevalence >0.75) and 1–3 less common subtypes (overall prevalence <0.5) (see Table S2). Some subtypes showed little variation in prevalence throughout the study (e.g., *T. velifera* and *T. velifera* B), whereas others exhibited oscillatory patterns (e.g., *T. mutans*, *T. mutans* MSD, and *T*. (sp.) buffalo) (Figure 3d). As prevalence is used to estimate transmission of parasites within a system (Hens et al., 2012), our findings suggest there may be variation in transmission between taxa, and within each taxon, over time.

**Figure 4:**
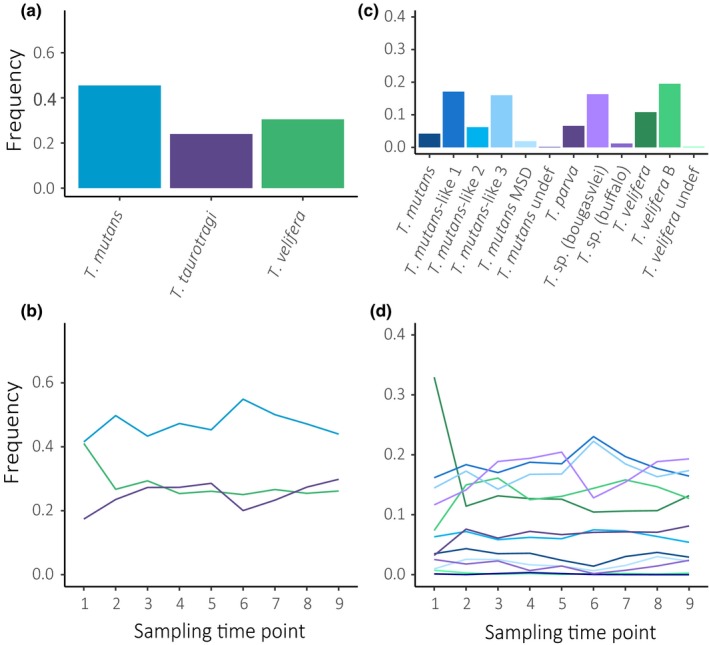
Frequencies of *Theileria* communities at a population level. Overall frequencies of *Theileria* spp. over the entire study and at each sampling time point for each clade (a, b, respectively) and subtype (c, d, respectively). Note: Colors for each taxon are identical in bar plots and line graphs

Clade‐level analysis of *Theileria* frequency indicates overall and temporal variation in frequency of clades (Figure 4a,b; Table S2) and subtypes (Figure 4c; Table S2). Notably, subtypes that occur at high prevalence throughout the study period (e.g., *T. mutans*‐like 1, *T. mutans*‐like 3, *T. velifera*, and *T. velifera* B) also occur at high frequencies; however, the variation in overall frequency between taxa is much more pronounced. As such, including frequency data provides a more informative depiction of population‐level parasite dynamics than prevalence alone. Frequency appears to remain somewhat constant for the majority of the subtypes with a few exceptions (Figure 4d). Interestingly, *T. mutans*‐like 1 and *T. mutans*‐like 3 appear to undergo synchronous fluctuations, whereas both *T. mutans*‐like 1 and *T. mutans*‐like 3 appear to undergo antagonistic fluctuations with *T*. (sp.) bougasvlei (Figure 3d).

#### 3.2.2 Individual patterns

**Figure 5:**
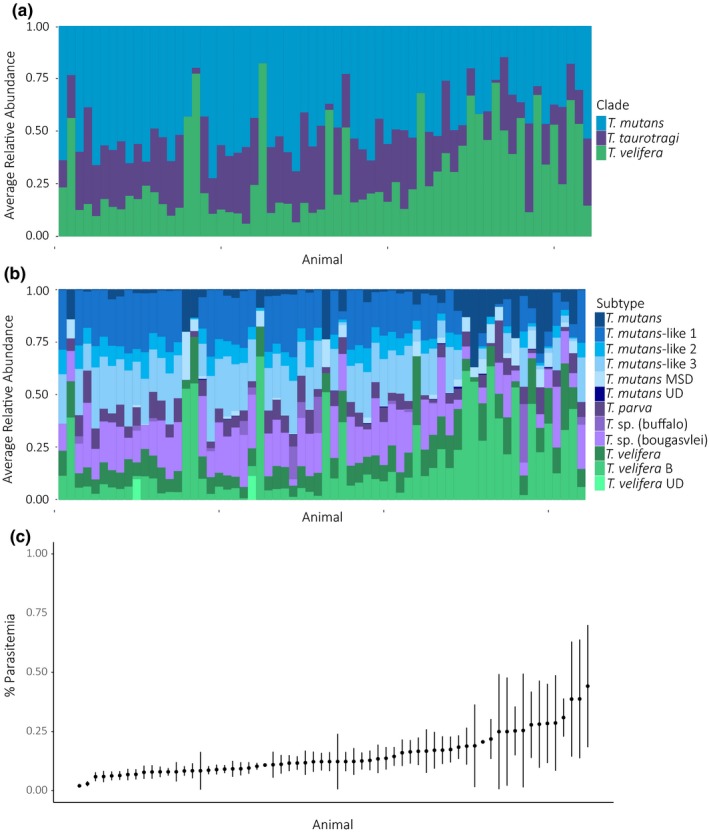
Parasitemia and variation in community composition at an individual level. (a) Averaged clade community composition for each animal. (b) Averaged subtype community composition. (c) Percent parasitemia (mean and *SE*) for each animal. The *y*‐axis extends from 0% to 1% (not 100%). Each figure is ordered from the animal with the lowest mean % parasitemia to the highest % parasitemia

We found that community composition was significantly different between hosts at the clade (Figure 5a; Table 1, PERMANOVA, *R* ^2^ = .66, *p*‐value < .001) and subtype level (Figure 5b; Table 2, PERMANOVA, *R* ^2^ = .723, *p*‐value < .001).

**Table 1:** PERMANOVA results table for clade community composition

	*df*	SS	MS	*F* statistic	*R* ^2^	*p*
Time	8	0.687	0.086	4.477	.030	<.001
Animal	65	15.077	0.232	12.092	.662	<.001
Residuals	366	7.021	0.019		.308	
Total	439	22.786			1.000	

Note

*p*‐values based on 999 permutations.

Abbreviations: animal, animal ID; *df*, degrees of freedom; MS, mean sum of squares; SS, sum of squares; time, sampling time point.

**Table 2:** PERMANOVA results table for subtype community composition

	*df*	SS	MS	*F* statistic	*R* ^2^	*p*
Time	8	1.374	0.172	5.552	.030	<.001
Animal	65	33.677	0.518	16.746	.726	<.001
Residuals	366	11.323	0.031		.244	
Total	439	46.374			1.000	

Note

*p*‐values based on 999 permutations.

Abbreviations: animal, animal ID; *df*, degrees of freedom; MS, mean sum of squares; SS, sum of squares; time, sampling time point.

Animals with high parasitemia appeared to have distinctly different communities than those with low parasitemia, both at the clade subtype level (Figure 5). The variance in parasitemia appeared to increase with mean parasitemia (Figure 5c). At the clade level, animals with higher average relative abundance of *T. velifera* had the highest mean and most variable parasitemia (Figure 5a,c). Similarly, at the subtype level, animals with a higher relative abundance of *T. velifera* B had the highest mean and the most variable parasitemia (Figure 5b,c). Notably, animals with higher mean parasitemia, and corresponding high parasitemia variance, also had higher average relative abundance of subtype *T. mutans*.

## 4 DISCUSSION

We utilized NGSA to investigate previously cryptic dynamics of *Theileria* communities in wild African buffalo at the herd and individual levels over a two‐year period.

We found that our methodology increased the breadth of data collected within our system, as we simultaneously identified three species clades, and twelve closely related *Theileria* subtypes, two of which had not previously been reported in our system (see Figure 2). We increased the depth of data collected by analyzing data at two taxonomic levels (species group and subtype) and established methodological framework to collect data at broader (genera: all *Theileria* and *Babesia* species) and narrower (genotype) taxonomic groupings (Figures 3, 4, 5). Finally, we increased the precision at which we were able to view community dynamics by obtaining relative abundance data for each taxon (Figures 4 and 5).

In particular, the increase in depth and precision enabled us to observe patterns not discernible using traditional analytical approaches. When analyzing our data at the clade level, we found uniformly high prevalence across individuals and over time (Figure 3a,b). These findings match Henrichs et al. (2016), which found African buffalo to be infected with the same species clades at all points in time and was thus unable to tease apart *Theileria* community dynamics due to the use of invariable, qualitative data at broad taxonomic levels. Subtype analyses revealed a much more dynamic system: subtypes varied in overall prevalence with a handful of subtypes remaining remarkably constant over time and others exhibiting synchronous and/or antagonist fluctuations in prevalence (Figure 3c,d). Variation among taxa and similarities in temporal trends at the clade and subtype level became even more distinct when analyzing the frequency, or relative abundance of each taxon, at the population level (Figure 4). Interestingly, examining subtype frequency revealed that only 1–2 subtypes drive dominance of species clades. Furthermore, the high frequency of *T*. sp. (bougasvlei), yet relatively low frequency of the *T. taurotragi* clade, highlights that examining data at coarse taxonomic levels may mask the effects of influential taxa within a system. Overall, variation in population patterns of each taxon suggests heterogeneous transmission within this genus, while synchronous and opposite patterns of abundance may point to significant interactions among *Theileria* subtypes—trends that future research can further investigate.

We found striking associations between mean parasitemia, parasitemia variance, and community composition (Figure 5). Our data visualization indicated that animals with higher mean parasitemia have, on average, conspicuously, higher relative abundances of the *T. velifera* species clade and lower relative abundances of the *T. mutans* species clade. Community composition reveals interesting patterns at the subtype level, albeit with additional nuances: trends observed in the *T. velifera* species groups appeared to be primarily driven by dominance of *T. verlifera* B; furthermore, animals with higher mean parasitemia had, on average, had lower relative abundances of *T. mutans* species groups but higher relative abundances of *T. mutans* (Figure 5b). Parasitemia has been negatively associated with host health outcomes (Asghar et al., 2011; Sol et al., 2003; Stjernman et al., 2008) as such *T. velifera* B and *T. mutans* may be the more pathogenic subtypes within this system. However, hosts may also be tolerant of *Theileria* (i.e., as parasitemia increases, host fitness remains constant; Råberg, Graham, & Read, 2009); in this case, parasitemia would not negatively correlate with host fitness or, perhaps, tolerance varies with community composition. Overall, our methods enable exploring how community composition influences host fitness with initial links to parasitemia offering interesting hypotheses regarding how fine scale in parasite community affects host health outcomes.

Importantly, the diversity of our communities is well supported by the existing literature. We detected almost all *Theileria* subtypes previously detected in southern KNP (Mans et al., 2016). We did not find *T*. sp. (sable), which has previously been reported in African buffalo in KNP (Henrichs et al., 2016). *T*. sp. (sable) may have been previously reported in African buffalo as the *T*. sp. (sable) RLB probe crosshybridizes with *T. velifera* (Mans et al., 2011). Mans et al. (2016) used amplicon sequencing, using the Roche 454 platform, to describe the prevalence of *Theileria* spp. in South Africa, but did not detect *T*. sp. (sable) in African buffalo. The absence of *T*. sp. (sable) within our study underlines how NGSA ameliorates specificity and sensitivity issues, such as crossreactivity, that plague alternative diagnostic tools. Notably, we found two species that have not been previously reported. Interestingly, these species, particularly the subtype most closely related to *T. velifera*, were detected in the same animal across multiple time points. We may have detected these species because we used very high read coverage (on average 69,407 ± 1,929 reads per sample).

When adapting our methods to other study systems, we encourage careful consideration of study design. For example, if using markers more variable than the 18S gene (e.g., more low frequency yet biologically important sequences) or addressing questions that necessitate the inclusion of low frequency sequences (e.g., mutation and evolution), we suggest running all samples in triplicate. We found the relative abundances of unique sequences within our samples were highly repeatable at a relative abundance of >1% (Appendix S2). However, during our replication experiment, we found a few low abundance sequences (<1%) that occurred in both replicates. Using duplicates or triplicates of all samples would allow researchers to differentiate between true low abundance sequences and noise, allowing for accurate reporting of genetic diversity within a population.

Overall, we found that using an NGS‐based approach allowed us to obtain data powerful enough to further our understanding of community dynamics in the *Theileria*—African buffalo system. Our dataset will enable us to explore a range of questions, including explicitly defining mechanistic links between parasite community and host health as well as community processes that alter pathogen persistence. Notably, the primers used for NGSA are conserved across all species of *Theileria* and *Babesia*, regardless of host species (Gubbels et al., 1999). Thus, this methodology can be used to study blood‐borne parasite communities of a broad range of host species, including the tick vector. We believe that, given the appropriate genetic marker, our workflow is readily adaptable to other disease systems. As exemplified by our study, the application of NGSA in disease ecology will exponentially increase our understanding of causes and consequences of variation in parasite communities in natural host populations**.**

## CONFLICT OF INTEREST

None declared.

## AUTHOR'S CONTRIBUTIONS

CKG helped with study design, data collection, led bioinformatics, phylogenetic, and community analyses as well as manuscript preparation. AVK helped with study design, bioinformatics, and phylogenetic analyses. RSH helped with bioinformatics analyses. MAS and MC contributed toward the development and analyses of the quantitative PCR. BRB managed and designed the field study and managed the team of scientists from OSU. BC contributed to study design and managed the team of scientists from the Pirbright. RBG supported the study through the provision of computing infrastructure, informatic support, and associated salaries (AVK and RSH). AEJ led field study design and managed the team of scientists from OSU and acted as mentor to CKG. AJ led study design of analytical (NGSA and qPCR) tool development and managed the team of scientists from UM as well as acted as mentor to CKG.

## ETHICAL APPROVAL

The study was conducted under South African Department of Agriculture, Forestry and Fisheries Section 20 permits Ref 12/11/1, ACUP project number 4478, Onderstepoort Veterinary Research Animal Ethics Committee project number 100261‐Y5, and the Kruger National Park Animal Care and Use Committee project number JOLAE1157‐12.

## Data Availability

Data available from GenBank. GenBank Accession Numbers: MK792966–MK92994.
