# Supplementary material for: Elucidating cryptic dynamics of Theileria communities in African buffalo using a high‐throughput sequencing informatics approach
Source: Ecol Evol. 2019 Dec 20;10(1):70–80. doi: 10.1002/ece3.5758 (PMC6972817; doi:10.1002/ece3.5758)
Supplement: Supplementary file 1 [file ECE3-10-70-s001.docx]

**Supporting information Appendix S1**. *In silico* mock community analysis.

First, using GenBank, a library of 63 previously published 18S *Theileria* sequences was compiled. An in-house Python script, looping an ART (V 3.19.15; Haung *et al.* 2012) script, was used to randomly select and generate FASTQ files from two *Theileria* species and three haplotypes per species 25 times. Illumina MiSeq forward and reverse read error profiles were averaged across 300 samples to obtain an error profile for each simulated FASTQ file. Each simulation iteration produced one major haplotype (high relative abundance) per species and two minor haplotypes (low relative abundance) per *Theileria* species. The collective mock community consisted of 25 simulated samples, with major haplotypes sampled at 35%, 47%, 48.5%, 49.25% and 49.625% relative abundances and minor haplotypes sampled at 10%, 5%, 2%, 1%, 0.5%, 0.25% and 0.125% relative abundances.

SeekDeep and DADA2 were run using default settings for Illumina MiSeq paired-end reads. For SeekDeep, FASTQ files from all samples were processed using a within-sample relative abundance cutoff of 0% and the Illumina MiSeq tag, allowing no mismatches. Within the SeekDeep pipeline, sequences that were marked as likely chimeric were removed. For DADA2, FASTQ files were processes using the denoise-paired command, trimming sequences at 20 base pairs. No additional parameters were included in the command. Relative abundance matrixes produced by each software were exported into R software (V. 3.4.3) and compared to known relative abundances using the Mantel test (Bray-Curtis distance measures) in the vegan package (Oksanen *et al.* 2018). For both software packages, Mantel correlation coefficient was > 0.99. Subsequently, the number of false haplotypes produced by each software was evaluated. Here, we define false haplotypes as sequences that were produced by the software which were not included in our mock community.

When evaluating haplotypes that occurred at > 0.1% relative abundance within a sample, SeekDeep produced no false haplotypes whereas DADA2 produced two false haplotypes; hence, SeekDeep was used for all further analyses.

Table S1. Layout of *in silico* mock community: Relative abundance of each sequence included in each simulated sample. Species 1 and species 2 varied between each sample.

| Sample | Relative abundance species 1.A (%) | Relative abundance species 1.B  (%) | Relative abundance species 1.C  (%) | Relative abundance species 2.A  (%) | Relative abundance species 2.B  (%) | Relative abundance species 2.C  (%) |
| --- | --- | --- | --- | --- | --- | --- |
| 1 | 35 | 10 | 5 | 35 | 10 | 5 |
| 2 | 35 | 10 | 5 | 35 | 10 | 5 |
| 3 | 35 | 10 | 5 | 35 | 10 | 5 |
| 4 | 35 | 10 | 5 | 35 | 10 | 5 |
| 5 | 35 | 10 | 5 | 35 | 10 | 5 |
| 6 | 47 | 2 | 1 | 47 | 2 | 1 |
| 7 | 47 | 2 | 1 | 47 | 2 | 1 |
| 8 | 47 | 2 | 1 | 47 | 2 | 1 |
| 9 | 47 | 2 | 1 | 47 | 2 | 1 |
| 10 | 47 | 2 | 1 | 47 | 2 | 1 |
| 11 | 48.5 | 1 | 0.5 | 48.5 | 1 | 0.5 |
| 12 | 48.5 | 1 | 0.5 | 48.5 | 1 | 0.5 |
| 13 | 48.5 | 1 | 0.5 | 48.5 | 1 | 0.5 |
| 14 | 48.5 | 1 | 0.5 | 48.5 | 1 | 0.5 |
| 15 | 48.5 | 1 | 0.5 | 48.5 | 1 | 0.5 |
| 16 | 49.25 | 0.5 | 0.25 | 49.25 | 0.5 | 0.25 |
| 17 | 49.25 | 0.5 | 0.25 | 49.25 | 0.5 | 0.25 |
| 18 | 49.25 | 0.5 | 0.25 | 49.25 | 0.5 | 0.25 |
| 19 | 49.25 | 0.5 | 0.25 | 49.25 | 0.5 | 0.25 |
| 20 | 49.25 | 0.5 | 0.25 | 49.25 | 0.5 | 0.25 |
| 21 | 49.625 | 0.25 | 0.125 | 49.625 | 0.25 | 0.125 |
| 22 | 49.625 | 0.25 | 0.125 | 49.625 | 0.25 | 0.125 |
| 23 | 49.625 | 0.25 | 0.125 | 49.625 | 0.25 | 0.125 |
| 24 | 49.625 | 0.25 | 0.125 | 49.625 | 0.25 | 0.125 |
| 25 | 49.625 | 0.25 | 0.125 | 49.625 | 0.25 | 0.125 |
