# Supplementary material for: Elucidating cryptic dynamics of Theileria communities in African buffalo using a high‐throughput sequencing informatics approach
Source: Ecol Evol. 2019 Dec 20;10(1):70–80. doi: 10.1002/ece3.5758 (PMC6972817; doi:10.1002/ece3.5758)
Supplement: Supplementary file 2 [file ECE3-10-70-s002.docx]

**Supporting information Appendix S2. Duplication study**

Prior to running FASTQ files from all samples through the bioinformatics pipeline, 10% of samples were run in duplicates in separate library assembly reactions and de-multiplexed, filtered and clustered on SeekDeep using the Illumina MiSeq tag and a within-sample relative abundance cutoff of 0.1%. Pairwise differences were assessed using the paired Wilcoxon signed-rank test in the software *R*. After increasing the relative abundance cutoff to 1%, there were no significant differences in the distribution of relative abundances within duplicate pairs (*P* > 0.05). To further confirm the repeatability of duplicates, an ordination was plotted after applying the within-sample 1% relative abundance cutoff using the *R* vegan package (SM Figure 2; non-metric multi-dimensional scaling, Bray-Curtis distance measures, stress = 0.07) (Oksanen *et al.* 2018).

**Supporting information 2 figure 1.** A non-metric multidimensional scaling (NDMS) plot for the ordination of distance between duplicates PCR of amplicons sequenced using next-generation sequencing. Each duplicate is a unique color and connected with a line. Duplicates are closer to each other in ordination space than other duplicates, particularly along the first axis. Additionally, for each pair of duplicates, a Wilcoxon sum signed-rank test was conducted to ensure duplicate relative abundances came from the same distribution (*P* > 0.05).
