# Supplementary material for: Elucidating cryptic dynamics of Theileria communities in African buffalo using a high‐throughput sequencing informatics approach
Source: Ecol Evol. 2019 Dec 20;10(1):70–80. doi: 10.1002/ece3.5758 (PMC6972817; doi:10.1002/ece3.5758)
Supplement: Supplementary file 3 [file ECE3-10-70-s003.docx]

**Supporting information Appendix S3. Calculation of % parasitemia**

(i) Plasmid construction

To prepare a template for the standard curve, a 178 bp region of the V4 hypervariable 18S ribosomal RNA fragment of *Theileria orientalis* was amplified using the forward (5’–GAC TTT GGT TCT ATT TTG TTG GA–3’) and the reverse RLBR (5’–TCT TCG ATC CCC TAA CTT TC–3’) oligonucleotide primers. The forward primer is the reverse complement of the reverse primer originally described by Sibeko *et al.* (2008) whereas the reverse primer was designed by Gubbels *et al.* (1999). The reagents and PCR conditions were optimised in a series of experiments; the final PCR was conducted in a 25 µL volume containing 10 mM Tris-HCl (pH 8.4), 50 mM KCl (Promega, Madison, WI, USA), 3.5 mM MgCl_2_, deoxynucleotide triphosphates (dNTPs; 200 µM each), primers (50 pmol each) and 1 U Go*Taq* polymerase (Promega) using the following protocol: 5 min at 95°C, followed by 35 cycles of 30 s at 95°C, 20 s at 60°C and 1 min at 72°C, followed by a final extension of 5 min at 72°C. PCR products were run on 3% (w/v) agarose gel-purified using the QIAquick Gel Extraction kit (Qiagen) and cloned into the pGEM®-T Easy Vector System (Promega) as per manufacturer’s instructions. Plasmid DNA was purified from transformed cells (JM109 competent cells, Promega) using Wizard® Plus SV Minipreps (Promega), quantified by spectrophotometer (Nanodrop 3000?) at 260 nm wavelength, and then subjected to bi-directional, automated Sanger sequencing using the same primers used in PCR. The quality of the sequences was assessed using the program Geneious Pro 2.0.10 (Kearse *et al*. 2012) and the specificity was confirmed with previously published 18S sequences of *T. orientalis*.

Molecular conversion calculations, based on size and base composition of the DNA fragment, were used to estimate the DNA copy number in each plasmid solution. A 10-fold serial dilution in molecular grade water (1x10^8^ copy number per reaction – 1x10^1^ copy number per reaction) was created for use as the standard curve.

(ii) qPCR validation

Reagents and conditions of the qPCR were optimised in a series of experiments. The final qPCR was conducted in 20 µL volume containing 10 µL of Dye Based Master Mix (Promega, Australia), 1 µL of each primer (10 pmol), 4 µL ddH_2_0 and 4 µL of DNA template using the following conditions: 5 min at 95 °C, followed by 40 cycles of 30 s at 95 °C, 30 s at 58 °C, 20 s at 72 °C, followed by a final extension of 5 min at 72 °C. Amplicon melt analysis was performed using temperature ramps of 0.3 °C between 70 and 99 °C. Each plasmid DNA dilution was run in triplicate and samples were run in duplicates. All reactions were prepared using the QIAgility (Qiagen) automated PCR setup system. qPCR reactions were run using a Rotor-gene Q (Qiagen) thermocycler. A positive (*T. orientalis*) and no-template controls were included in each assay. The specificity of the assay was based on the analyses of the conventional and normalised high resolution melt (HRM) curves of amplicons derived from the positive control samples, and *Theileria* was assigned in test samples based on mean HRM temperature. Selected qPCR products were cloned using methods as above and sequenced.

The analytical sensitivity was determined using a 10-fold serial dilution of plasmids whereas the analytical specificity was assessed using DNAs of *T. orientalis*, *T. velifera, T. mutans, Babesia bovis, B. bigemina, Anaplasma marginale, A. centrale, A. platys and Ehrlichia canis*. Assay repeatability was confirmed by running each standard curve in triplicate across three separate qPCR reactions over two days. Inter-assay variability was determined by comparing amplification efficiency (efficiency = -1 + 10 ^(-1/slope)^, the slope is of the log-linear relationship between DNA copy number and the cycle threshold (*C_T_*) values) and correlation coefficient (R^2^) across plates. Additionally, for each plate, the mean *C_T_* value was calculated for each dilution. The coefficient of variation (CV: standard deviation/mean) of mean *C_T_* value per standard curve dilution was calculated across plates. Intra-assay variability was assessed by calculating the CV of *C_T_* values for each dilution within each plate.

(iii) qPCR validation results

The identity of individual products was confirmed by sequencing, and no product was amplified when the DNA template was used from *A. marginale, A. centrale, A. platys* or *E. canis*. When comparing across plates, for *T. orientalis*, mean amplification efficiency was 0.93±0.02, mean R^2^ was 0.987±0.005 and mean CV was 8.81±0.49 (range 5.47-16.13). Intra-assay CV values for *T. orientalis* ranged from 0.0017-0.0218 with a mean of 0.0112 (SE±0.0003).

(iv) Calculation of parasitaemia

We calculated % parasitaemia (parasitaemia, hereafter) from the qPCR result by following a method previously described by Pienaar *et al.* (2011). Volume of whole blood used in the qPCR assay was calculated by multiplying the proportion of DNA extract used in the assay by volume of whole blood used in the DNA extraction. Subsequently, the number of red blood cells in each reaction was then calculated by multiplying volume (number of microliters) of whole blood used in each reaction by the number of red blood cells per microliter. As the 18S gene of *Theileria* is believed to have two copies per genome (Gardner *et al.* 2005; Hayashida *et al.* 2012; Kappmeyer *et al.* 2012; Pain *et al.* 2005), copy number per sample (calculated from mean of duplicates) was divided by two to obtain the number of parasites per reaction. Parasites in each qPCR reaction per sample was divided by red blood cells per reaction and multiplied by 100 to obtain % parasitemia (i.e. parasitemia).

(v) Parasitaemia summary statistics

Following the establishment of qPCR assay, a total of 440 (out of 443) samples were successfully amplified, and the cloning and sequencing of selected qPCR amplicons confirmed their identity as *Theileria* spp. Parasitaemia ranged from 0.001% - 1.151%; mean parasitemia was 0.126% (SE ± 0.008).

**Supporting information 3 fig 1.** qPCR melt curves for *Theileria* spp. and *Babesia* spp. positive controls
