# Supplementary material for: Elucidating cryptic dynamics of Theileria communities in African buffalo using a high‐throughput sequencing informatics approach
Source: Ecol Evol. 2019 Dec 20;10(1):70–80. doi: 10.1002/ece3.5758 (PMC6972817; doi:10.1002/ece3.5758)
Supplement: Supplementary file 4 [file ECE3-10-70-s004.docx]

**Supporting information Table S1.** Pairwise distances (number of base pairs) between each unique sequence; sequences are grouped by taxa.

|  | 1 | 2 | 3 | 4 | 5 | 6 | 7 | 8 | 9 | 10 | 11 | 12 | 13 | 14 | 15 | 16 | 17 | 18 | 19 | 20 | 21 | 22 | 23 | 24 | 25 | 26 | 27 | 28 | 29 |
| --- | --- | --- | --- | --- | --- | --- | --- | --- | --- | --- | --- | --- | --- | --- | --- | --- | --- | --- | --- | --- | --- | --- | --- | --- | --- | --- | --- | --- | --- |
| 1.MK792977 |  | 1 | 2 | 15 | 18 | 18 | 23 | 21 | 22 | 18 | 20 | 19 | 22 | 21 | 51 | 52 | 52 | 50 | 52 | 54 | 54 | 53 | 53 | 54 | 54 | 56 | 53 | 56 | 57 |
| 2.MK792978 | 1 |  | 1 | 16 | 19 | 19 | 24 | 22 | 23 | 19 | 19 | 18 | 23 | 20 | 51 | 52 | 52 | 50 | 52 | 54 | 54 | 53 | 53 | 54 | 54 | 57 | 54 | 57 | 58 |
| 3.MK792985 | 2 | 1 |  | 15 | 18 | 18 | 23 | 21 | 22 | 18 | 20 | 19 | 24 | 21 | 50 | 51 | 51 | 49 | 51 | 53 | 53 | 52 | 52 | 53 | 53 | 56 | 53 | 56 | 57 |
| 4.MK792976 | 15 | 16 | 15 |  | 10 | 10 | 14 | 12 | 13 | 12 | 16 | 17 | 19 | 16 | 43 | 44 | 44 | 42 | 44 | 43 | 43 | 40 | 40 | 41 | 41 | 49 | 45 | 49 | 50 |
| 5.MK792968 | 18 | 19 | 18 | 10 |  | 2 | 12 | 11 | 11 | 15 | 20 | 21 | 25 | 20 | 47 | 48 | 46 | 46 | 48 | 49 | 49 | 46 | 46 | 47 | 47 | 49 | 45 | 49 | 50 |
| 6.MK792992 | 18 | 19 | 18 | 10 | 2 |  | 13 | 11 | 12 | 17 | 20 | 21 | 25 | 20 | 47 | 48 | 46 | 46 | 48 | 49 | 49 | 46 | 46 | 47 | 47 | 47 | 43 | 47 | 48 |
| 7.MK792972 | 23 | 24 | 23 | 14 | 12 | 13 |  | 2 | 1 | 13 | 17 | 18 | 21 | 17 | 48 | 48 | 47 | 47 | 49 | 48 | 48 | 47 | 47 | 48 | 48 | 54 | 50 | 54 | 55 |
| 8.MK792973 | 21 | 22 | 21 | 12 | 11 | 11 | 2 |  | 1 | 12 | 15 | 16 | 19 | 15 | 46 | 46 | 45 | 45 | 47 | 47 | 47 | 46 | 46 | 47 | 47 | 53 | 49 | 53 | 54 |
| 9.MK792975 | 22 | 23 | 22 | 13 | 11 | 12 | 1 | 1 |  | 12 | 16 | 17 | 20 | 16 | 47 | 47 | 46 | 46 | 48 | 48 | 48 | 47 | 47 | 48 | 48 | 54 | 50 | 54 | 55 |
| 10.MK792986 | 18 | 19 | 18 | 12 | 15 | 17 | 13 | 12 | 12 |  | 18 | 19 | 20 | 18 | 49 | 50 | 50 | 48 | 50 | 50 | 50 | 49 | 49 | 50 | 50 | 57 | 54 | 57 | 58 |
| 11.MK792970 | 20 | 19 | 20 | 16 | 20 | 20 | 17 | 15 | 16 | 18 |  | 1 | 8 | 1 | 47 | 48 | 48 | 46 | 48 | 47 | 47 | 46 | 46 | 47 | 47 | 52 | 50 | 53 | 54 |
| 12.MK792980 | 19 | 18 | 19 | 17 | 21 | 21 | 18 | 16 | 17 | 19 | 1 |  | 9 | 2 | 48 | 49 | 49 | 47 | 49 | 48 | 48 | 47 | 47 | 48 | 48 | 53 | 51 | 54 | 55 |
| 13.MK792990 | 22 | 23 | 24 | 19 | 25 | 25 | 21 | 19 | 20 | 20 | 8 | 9 |  | 9 | 46 | 47 | 47 | 45 | 47 | 46 | 46 | 45 | 45 | 46 | 46 | 55 | 53 | 56 | 57 |
| 14.MK792994 | 21 | 20 | 21 | 16 | 20 | 20 | 17 | 15 | 16 | 18 | 1 | 2 | 9 |  | 47 | 48 | 48 | 46 | 48 | 47 | 47 | 46 | 46 | 47 | 47 | 52 | 50 | 53 | 54 |
| 15.MK792969 | 51 | 51 | 50 | 43 | 47 | 47 | 48 | 46 | 47 | 49 | 47 | 48 | 46 | 47 |  | 1 | 1 | 1 | 1 | 9 | 9 | 11 | 10 | 10 | 12 | 44 | 40 | 44 | 45 |
| 16.MK792979 | 52 | 52 | 51 | 44 | 48 | 48 | 48 | 46 | 47 | 50 | 48 | 49 | 47 | 48 | 1 |  | 2 | 2 | 2 | 10 | 10 | 12 | 11 | 11 | 13 | 45 | 41 | 45 | 46 |
| 17.MK792982 | 52 | 52 | 51 | 44 | 46 | 46 | 47 | 45 | 46 | 50 | 48 | 49 | 47 | 48 | 1 | 2 |  | 2 | 2 | 10 | 10 | 12 | 11 | 11 | 13 | 44 | 40 | 44 | 45 |
| 18.MK792989 | 50 | 50 | 49 | 42 | 46 | 46 | 47 | 45 | 46 | 48 | 46 | 47 | 45 | 46 | 1 | 2 | 2 |  | 2 | 10 | 10 | 12 | 11 | 11 | 13 | 45 | 41 | 45 | 46 |
| 19.MK792991 | 52 | 52 | 51 | 44 | 48 | 48 | 49 | 47 | 48 | 50 | 48 | 49 | 47 | 48 | 1 | 2 | 2 | 2 |  | 10 | 10 | 12 | 11 | 11 | 13 | 45 | 41 | 45 | 46 |
| 20.MK792981 | 54 | 54 | 53 | 43 | 49 | 49 | 48 | 47 | 48 | 50 | 47 | 48 | 46 | 47 | 9 | 10 | 10 | 10 | 10 |  | 1 | 3 | 4 | 2 | 4 | 41 | 36 | 40 | 41 |
| 21.MK792988 | 54 | 54 | 53 | 43 | 49 | 49 | 48 | 47 | 48 | 50 | 47 | 48 | 46 | 47 | 9 | 10 | 10 | 10 | 10 | 1 |  | 3 | 4 | 2 | 4 | 41 | 36 | 40 | 41 |
| 22.MK792971 | 53 | 53 | 52 | 40 | 46 | 46 | 47 | 46 | 47 | 49 | 46 | 47 | 45 | 46 | 11 | 12 | 12 | 12 | 12 | 3 | 3 |  | 1 | 1 | 1 | 38 | 33 | 37 | 38 |
| 23.MK792983 | 53 | 53 | 52 | 40 | 46 | 46 | 47 | 46 | 47 | 49 | 46 | 47 | 45 | 46 | 10 | 11 | 11 | 11 | 11 | 4 | 4 | 1 |  | 2 | 2 | 38 | 33 | 37 | 38 |
| 24.MK792984 | 54 | 54 | 53 | 41 | 47 | 47 | 48 | 47 | 48 | 50 | 47 | 48 | 46 | 47 | 10 | 11 | 11 | 11 | 11 | 2 | 2 | 1 | 2 |  | 2 | 39 | 34 | 38 | 39 |
| 25.MK792993 | 54 | 54 | 53 | 41 | 47 | 47 | 48 | 47 | 48 | 50 | 47 | 48 | 46 | 47 | 12 | 13 | 13 | 13 | 13 | 4 | 4 | 1 | 2 | 2 |  | 38 | 33 | 37 | 38 |
| 26.MK792966 | 56 | 57 | 56 | 49 | 49 | 47 | 54 | 53 | 54 | 57 | 52 | 53 | 55 | 52 | 44 | 45 | 44 | 45 | 45 | 41 | 41 | 38 | 38 | 39 | 38 |  | 10 | 2 | 3 |
| 27.MK792987 | 53 | 54 | 53 | 45 | 45 | 43 | 50 | 49 | 50 | 54 | 50 | 51 | 53 | 50 | 40 | 41 | 40 | 41 | 41 | 36 | 36 | 33 | 33 | 34 | 33 | 10 |  | 8 | 8 |
| 28.MK792967 | 56 | 57 | 56 | 49 | 49 | 47 | 54 | 53 | 54 | 57 | 53 | 54 | 56 | 53 | 44 | 45 | 44 | 45 | 45 | 40 | 40 | 37 | 37 | 38 | 37 | 2 | 8 |  | 1 |
| 29.MK792974 | 57 | 58 | 57 | 50 | 50 | 48 | 55 | 54 | 55 | 58 | 54 | 55 | 57 | 54 | 45 | 46 | 45 | 46 | 46 | 41 | 41 | 38 | 38 | 39 | 38 | 3 | 8 | 1 |  |
