# Supplementary material for: Elucidating cryptic dynamics of Theileria communities in African buffalo using a high‐throughput sequencing informatics approach
Source: Ecol Evol. 2019 Dec 20;10(1):70–80. doi: 10.1002/ece3.5758 (PMC6972817; doi:10.1002/ece3.5758)
Supplement: Supplementary file 5 [file ECE3-10-70-s005.docx]

**Supporting information Table S2: Table of prevalence and frequency for each clade, subtype and unique sequence.**

|  | **Parasite** | | | | **Parasite subtype** | | | | | **Consensus sequences** | | | | |
| --- | --- | --- | --- | --- | --- | --- | --- | --- | --- | --- | --- | --- | --- | --- |
| clade | No. sample | Prevalence | No. reads | Frequency | subtype | No. samples | Prevalence | No. reads | Frequency | sequence | No. samples | Prevalence | No. reads | Frequency |
|  |  |  |  |  |  |  |  |  |  |  |  |  |  |  |
| T. mutans | 435 | 0.989 | 14881605 | 0.455 | T. mutans | 226 | 0.514 | 1365312 | 0.042 | MK792976 | 226 | 0.514 | 1365312 | 0.042 |
|  |  |  |  |  | T. mutans MSD | 111 | 0.252 | 617170 | 0.019 | MK792977 | 108 | 0.245 | 337939 | 0.01 |
|  |  |  |  |  |  |  |  |  |  | MK792978 | 87 | 0.198 | 228888 | 0.007 |
|  |  |  |  |  |  |  |  |  |  | MK792985 | 30 | 0.068 | 50343 | 0.002 |
|  |  |  |  |  | T. mutans-like 1 | 392 | 0.891 | 5600756 | 0.171 | MK792992 | 4 | 0.009 | 4441 | 0 |
|  |  |  |  |  |  |  |  |  |  | MK792968 | 393 | 0.893 | 5596315 | 0.171 |
|  |  |  |  |  | T. mutans-like 2 | 387 | 0.880 | 2034428 | 0.062 | MK792970 | 388 | 0.882 | 1941601 | 0.059 |
|  |  |  |  |  |  |  |  |  |  | MK792980 | 70 | 0.159 | 78515 | 0.002 |
|  |  |  |  |  |  |  |  |  |  | MK792990 | 7 | 0.016 | 8476 | 0 |
|  |  |  |  |  |  |  |  |  |  | MK792994 | 2 | 0.005 | 5836 | 0 |
|  |  |  |  |  | T. mutans-like 3 | 375 | 0.852 | 5236552 | 0.16 | MK792972 | 374 | 0.850 | 2381203 | 0.073 |
|  |  |  |  |  |  |  |  |  |  | MK792973 | 371 | 0.843 | 2012109 | 0.061 |
|  |  |  |  |  |  |  |  |  |  | MK792975 | 262 | 0.595 | 843240 | 0.026 |
|  |  |  |  |  | T. mutans-like undefined | 20 | 0.045 | 27387 | 0.001 | MK792986 | 20 | 0.045 | 27387 | 0.001 |
|  |  |  |  |  |  |  |  |  |  |  |  |  |  |  |
| T. taurotragi | 410 | 0.932 | 7870959 | 0.24 | T. parva | 379 | 0.861 | 2146789 | 0.066 | MK792971 | 379 | 0.861 | 1991976 | 0.061 |
|  |  |  |  |  |  |  |  |  |  | MK792983 | 61 | 0.139 | 92841 | 0.003 |
|  |  |  |  |  |  |  |  |  |  | MK792984 | 41 | 0.093 | 58853 | 0.002 |
|  |  |  |  |  |  |  |  |  |  | MK792993 | 4 | 0.009 | 3119 | 0 |
|  |  |  |  |  | T. sp. bougasvlei | 387 | 0.880 | 5320496 | 0.163 | MK792969 | 388 | 0.882 | 5062700 | 0.155 |
|  |  |  |  |  |  |  |  |  |  | MK792979 | 72 | 0.164 | 120269 | 0.004 |
|  |  |  |  |  |  |  |  |  |  | MK792982 | 63 | 0.143 | 113107 | 0.003 |
|  |  |  |  |  |  |  |  |  |  | MK792989 | 8 | 0.018 | 16811 | 0.001 |
|  |  |  |  |  |  |  |  |  |  | MK792991 | 7 | 0.016 | 7609 | 0 |
|  |  |  |  |  | T. sp. buffalo | 68 | 0.155 | 403674 | 0.012 | MK792981 | 69 | 0.157 | 371345 | 0.011 |
|  |  |  |  |  |  |  |  |  |  | MK792988 | 12 | 0.027 | 32329 | 0.001 |
|  |  |  |  |  |  |  |  |  |  |  |  |  |  |  |
| T. velifera | 439 | 0.998 | 9974935 | 0.305 | T. velifera | 438 | 0.995 | 3541422 | 0.108 | MK792966 | 439 | 0.998 | 3541422 | 0.108 |
|  |  |  |  |  | T. velifera B | 416 | 0.945 | 6367970 | 0.195 | MK792967 | 401 | 0.911 | 3311741 | 0.101 |
|  |  |  |  |  |  |  |  |  |  | MK792974 | 300 | 0.682 | 3056229 | 0.093 |
|  |  |  |  |  | T. velifera-like undefined | 11 | 0.025 | 65543 | 0.002 | MK792987 | 11 | 0.025 | 65543 | 0.002 |
